# Robot-assisted laparoscopic bladder diverticulectomy and greenlight laser anatomic vaporization of the prostate

**DOI:** 10.1590/S1677-5538.IBJU.2017.0016

**Published:** 2018

**Authors:** Luca Cindolo, Manuela Ingrosso, Michele Marchioni, Ambra Rizzoli, Francesco Berardinelli, Luigi Schips

**Affiliations:** 1 Department of Urology, ASL Abruzzo 2, Chieti, Italy; 2 Department of Urology, SS Annunziata Hospital, “G. D'Annunzio” University of Chieti, Chieti, Italy

## INTRODUCTION

Many mini-invasive techniques were proposed to treat bladder diverticula due to chronic bladder outlet obstruction, however none of them combined GreenLight^®^ laser 180W (180W-GL) technology with robot-assisted bladder diverticulectomy.

## PRESENTATION

A 62 years old man with a retentive bladder diverticulum was placed in lithotomy position and an anatomic PVP was performed localizing the capsule at the apex of the adenoma by carrying out a bilateral incision lateral to the verumontanum, using the tip of the shaft of the resectoscope to push laterally. A careful blunt mechanical dissection of the plane with the resectoscope was followed toward the bladder neck at 6 o'clock and a vaporization of all the dissected tissue was made by firing the laser upwards from below the adenoma. The procedure was completed vaporizing laterally in both sides and anteriorly with the exact knowledge of the depth of the capsule. Two guide wires were placed in left ureter and diverticulum. A 20ch three-ways-catheter was placed. In supine position five trocars were placed. A 23 Trendelemburg-position was given. The diverticulum was isolated and dissected at the neck level. Detrusor muscle layer was closed in double watertight suture. Reperitonealization was performed over a drain.

## RESULTS

Operative time was 230 minutes. Blood loss was 50mL.

The catheter was removed on tenth post-operative day without complication. At 6 months, postvoid residual urine volume was 60ml with satisfactory stream (Qmax 21.8ml/s)

## CONCLUSIONS

Da Vinci robot and 180W-GL laser allow a mini-invasive BPH complications management, as bladder diverticula, permitting a rapid discharge.

## ARTICLE INFO

**Available at: http://www.intbrazjurol.com.br/video-section/20170016_Cindolo_et_al/**

**Int Braz J Urol. 2018; 44 (Video #4): 403-4**

